# On the Destruction of Non-Vascular Nœvi by the Vienna Caustic

**Published:** 1856-04

**Authors:** M. Chassaignac


*On the Destruction of Non-vascular Nævi by the Vienna Caustic.* By M. Chassaignac.

(Gaz. des. Hopitaux, Nos. 123 and 124, 1855.)

The pigmentary stains that have often been confounded with erectile tumors, under the common term of *nævi materni*, have been made the subject of special study by M. Chassaignac. He has shown that so far from being congenital, as generally believed, they do not appear, in the immense majority of cases, until after birth, and in many not until an advanced period of life. They do not consist solely in an accumulation of pigmentary matter, there existing, in a great number of instances, besides this, an appreciable quantity of a tissue he terms "fungoid." He distinguishes these merely pigmentary deposits from erectile tumors, which they often resemble, by observing the effects of the pressure of the finger, this rendering an erectile discoloration pale, while it effects no change of color in the pigmentary one. For treating this affection, M. Chassaignac regards the Vienna caustic as the best means, this inducing a dry eschar without suppuration, which leaves after its fall a smooth even cicatrix, that is moveable over the parts it covers, and differs little in color from the surrounding integuments.

The procedure is only applicable to slight thicknesses of living tissues. As soon as an eschar has been produced by the caustic sufficiently thick for the object in view, its surface is washed with a little vinegar and water, completely dried, and then it is covered with a piece of very supple amadon, cut so as to fit it exactly. If the access of all moisture be prevented, the amadon becomes so identified with the eschar that it falls off only with it, while the latter is not detached until the tissues subjacent to it are completely cicatrized. The intimate adhesion of the amadon is a *sine qua non* of success. M. Chassaignac maintains that all these spots, whatever their size or position, may be entirely removed by successive partial cauterisations of this kind, although the procedure, where they are large, exacts much time and patience. As an instance of this, he relates a case in which a nævus, the size of a crown piece, situated on the forehead, was attacked five times during seven months, the caustic being applied for from three to eight minutes, according to the different thickness presented, the application causing very considerable pain for some hours. A bandage was at first applied to retain the amadon, but after a few hours this was not required. The amodon came off with the esshar in from one to two months, leaving cicatrisation complete.

In a recent communication to the Paris Society of Surgery, M. Leclerc related a case of *true erectile tumor*, occuring in a child, which was successfully treated by the external applications three times a day of the *perchloride of iron*. It was continued during two months, giving rise to no pain or irritation whatever. It was long before any change occurred in the tumor, and its diminution was very gradual. The perchloride has already been several times resorted to in France, but only by injecting it into the vascular tissue ot the tumor, or by applying it after prior blistering.—*Med. Chir. Review*.

